# Characterization of resistance to last-line therapeutic molecules and genetic diversity of Klebsiella spp. strains involved in urinary tract infections in women: the case of Hôpital d’Instruction des Armées Omar Bongo Ondimba

**DOI:** 10.1099/jmm.0.002190

**Published:** 2026-08-27

**Authors:** Evrard Mayombo Ngoussou, Annicet Clotaire Dikoumba, Mundunge Mambu, Rolande Mabika Mabika, Ornella Zong Minko, Franck Mounioko, Léonce Fauster Ondjiangui, Rachel Moyen, Jean Fabrice Yala

**Affiliations:** 1 Laboratoire de Bactériologie Unité de Recherche d’Analyses Médicales, Centre Interdisciplinaire de Recherches Médicales de Franceville, BP: 769, Franceville, Gabon; 2 Unité Mixte de Recherche, Centre Interdisciplinaire de Recherches Médicales Franceville, BP: 769, Franceville, Gabon; 3 Laboratoire National, Libreville, Gabon; 4 Laboratoire de Biologie Moléculaire et Cellulaire, l’Université de Sciences et Techniques MASUKU, BP: 067, Franceville, Gabon; 5 Unité de Recherche d’Ecologie en Santé, Centre Interdisciplinaire de Recherches Médicales de Franceville, BP: 769, Franceville, Gabon; 6 Laboratoire de Biologie Moléculaire et Cellulaire, Faculté de Sciences et Techniques, Université Marien Ngouabi, BP: 69, Brazzaville, Republic of Congo

**Keywords:** enterobacterial repetitive intergenic consensus PCR (ERIC-PCR), female patients, *Klebsiella* spp., resistance genes, urinary tract infection (UTI)

## Abstract

**Introduction.** *Klebsiella* spp. are the second most common uropathogens after *Escherichia coli* and are globally recognized for their high morbidity, driven by their significant ability to develop multi-drug resistance against a wide range of antimicrobials.

**Hypothesis/Gap Statement.** In Central Africa, and particularly in Gabon, there is a significant lack of data regarding the prevalence and genetic diversity of uropathogenic *Klebsiella* spp. resistant to last-line antibiotics involved in urinary tract infections (UTIs) in women.

**Aim.** This study aims to characterize the resistance of *Klebsiella* spp. involved in UTIs in women to last-line therapeutic molecules and to explore their genetic diversity through enterobacterial repetitive intergenic consensus PCR (ERIC-PCR) profiling.

**Methodology.** The germs were isolated on Urilines biphasic slides. Identification and susceptibility profiles were performed using the API 20E, ATB Ur and VITEK^®^ 2 COMPACT. Molecular research of carbapenemase genes (*bla*_KPC_, *bla*_IMP_ and *bla*_*VIM*_) and resistance to fosfomycin (*fos*A, *fos*B, *fos*C, *glpT* and *uhpT*) was made by conventional PCR, and genetic diversity was screened using ERIC-PCR.

**Results.** Of 343 patients, 60.64% (208 out of 343) were women, with a prevalence of UTIs of 43.75% (91 out of 208). The involvement of *Klebsiella* spp. uropathogens was 29.03% (27 out of 93) against 53.76% (50 out of 93) of other enterobacteria. *Klebsiella* spp. strains showed high levels of resistance to third-generation cephalosporins (55.56%), fourth-generation cephalosporins and fosfomycin (40.74%), average levels of resistance to third-generation fluoroquinolones (25.92%) and low levels of resistance to carbapenems (7.41%). A total of 81.82% (18 out of 22) of *Klebsiella* spp. strains possess antibiotic resistance genes, *bla*_KPC_ (40.0%) and *fos*A (36.0%), respectively. The study of genetic diversity by interpretation of ERIC-PCR profiles shows 19 distinct banding patterns and 2 repeated profiles, all divided into 9 clusters.

**Conclusion.** Uropathogenic *Klebsiella* spp. isolated from women have high rates of resistance to last-line antibiotics, with the exception of imipenem. They exhibit diverse ERIC-PCR profiles and possess *bla*_KPC_ and *fos*A. These findings provide important preliminary surveillance data for the region.

## Introduction

Urinary tract infection (UTI) is an inflammation caused by the presence and growth of micro-organisms in the urinary tract and is defined by a minimum bacteriuria level of 10^4^ c.f.u. ml^−1^ with significant pyuria [1]. UTIs are among the most common nosocomial and community bacterial infections with around 150 million people affected worldwide each year and costing more than 6 billion dollars in treatment and affecting all age groups [2]. Their incidence in women is much higher than in men [3, 4]. This difference can be explained by the fact that bacteria live around the urethra and colonize the bladder, and the shorter distance to the bladder in women makes it easier for bacteria to reach it. In addition, the urogenital manipulations associated with daily life or medical interventions in women facilitate the movement of bacteria towards the urethra, as the urethral opening in women is close to the rectum [5]. However, even in women, they depend on physiological conditions [6]. Indeed, the prevalence of UTIs is higher in pregnant women than in non-pregnant women [7]. Obviously, UTI is one of the most common infections during pregnancy, diagnosed in 50–60% of all pregnancies [8].

*Enterobacteriaceae* are the most numerous, accounting for around 75% of isolates responsible for these infections [9]. In clinical and human pathology, they are the bacterial family responsible for the most frequent infections, which may be community-acquired or nosocomial, and may have the highest mortality rate [10]. Among uropathogens, *Escherichia coli* is responsible for 70–80% of UTIs, followed by *Proteus mirabilis* and *Klebsiella* spp*.* [11]. *Klebsiella* spp*.* are commensal pathogens normally found in the flora of the nose, throat, skin and intestinal tract of healthy people but cause infections such as pneumonia, UTIs and blood infections [12]. In addition to being responsible for several infections, they possess a number of pathogenicity factors, such as pili, capsule, LPS and iron transporters (siderophores) [13]. Apparently, these infections have the particularity of having a high morbidity and mortality rate, attributed to the hypervirulence of the *Klebsiella* strains associated with them [14].

UTIs are treated with antibiotics, including first-line and last-line drugs such as beta-lactam antibiotics, quinolones and fosfomycin [15]. However, the resistance of enterobacteria to antibiotics, particularly the uropathogenic *E. coli* and *Klebsiella*, has increased considerably due to the high level of empirical use of these molecules [16]. Changes in the susceptibility patterns of pathogenic bacteria isolated from UTIs highlight the need for epidemiological surveillance to generate information that can be used in patient management. This is all the more important given that a number of studies have revealed a high diversity of profiles in *Klebsiella* spp.

Conventional bacteriology techniques are unable to highlight the genetic diversity of bacterial isolates. This requires the use of molecular typing techniques. These techniques are valuable tools used to determine the grouping of *Klebsiella* spp*.* and to obtain information on the distribution of their genetic profiles. In this study, we used enterobacterial repetitive intergenic consensus PCR (ERIC-PCR). ERIC-PCR is based on genetic fingerprinting, due to the presence in the bacterial genome of multiple copies of conserved consensus sequences, in order to compare the similarity of banding profiles between bacterial isolates and thus enable their epidemiological surveillance [17]. ERIC-PCR remains one of the reference methods for characterizing the genetic diversity of enterobacteria, due to its simplicity and speed, its high discriminatory power and its lower cost compared with other molecular typing methods [18].

In Gabon, there are very few data on the prevalence of *Klebsiella* spp*.* UTIs in women. In addition, the genetic diversity of *Klebsiella* spp*.* strains has not yet been documented in Gabon. With a view to providing additional data on this problem, this study aims to characterize the genetic basis of resistance of these strains to last-line therapeutic antibiotics in relation to the diversity of their ERIC-PCR profiles.

## Methods

### Type and population of study

This was a cross-sectional descriptive study. Samples were collected in the medical analysis laboratory of the Omar Bongo Ondimba Army Training Hospital (HIAOBO) from 20 March to 26 May 2023. The samples collected were analysed in the bacteriology laboratory of the Interdisciplinary Center for Medical Research of Franceville (CIRMF).

The patients in this study were selected exhaustively. All hospitalized and/or ambulatory female patients who had undergone a urine cytobacteriological examination at the HIAOBO medical analysis laboratory and consented to participate were included. The participants’ socio-demographic data were collected using a survey form completed by the principal investigator in their presence. Age, sex, level of study, marital status, medical history, history of UTIs and antibiotic usage were all recorded on the form.

### Isolation and identification of *Klebsiella* strains

Any involved germs were isolated on biphasic slides (URILINE, bioMérieux) coated with Cystine–Lactose–Electrolyte-Deficient and MacConkey medium. The slides were immersed in the urine samples, drained to remove excess urine and incubated for 18 to 24 h at 37 °C. Cultures with a urine germ count ≥10^5^ c.f.u. ml^−1^, monomorphic and/or polymorphic with no more than two colonies, were considered positive and selected for further study.

Isolated germs were identified by two methods: biochemical identification using API 20E systems (bioMérieux, Marcy-l’Étoile) and/or the VITEK compact 2 automated system (bioMérieux), after orientation tests had been carried out. Bacterial suspensions of 0.5 McFarland opacity were prepared with 3 ml of sterile saline solution. These were then inoculated into cups according to the manufacturer’s instructions and incubated at 37 °C for 18–24 h. API 20E systems were read using bioMérieux’s mini analytical profile index (API) reader. For those identified by Vitek 2 compact, the inoculi and Gram-negative identification cards were placed in a cassette according to the manufacturer’s instructions.

### Extraction of total DNA from *Klebsiella* spp.

Total DNA extraction of *Klebsiella* spp*.* uropathogens was performed using the Invitrogen^™^ Mini kit RNA/DNA PureLink^™^, whose instructions were adapted. Seven to ten colonies isolated on Trypticase Soy agar were picked and washed in 200 µl of ultrapure water. Next, 200 µl of lysis buffer and 25 µl of 20 mg ml^−1^ proteinase K concentrate were added and homogenized. The preparation was then incubated at 56 °C for 1 h. After incubation, 250 µl of cold ethanol was added to the lysate and left at room temperature for 5 min. Finally, 650 µl of the mixture was transferred to collection tubes and centrifuged at 6,800 ***g*** for 10 min. The product was then washed twice with 300 µl of WII wash buffer and centrifuged at 6,800 ***g*** for 5 min. A total of 100 µl of sterile RNase water was added, and the preparations were centrifuged at maximum speed for 15 min. The DNAs obtained were stored at −20 °C until use.

### Phenotypic and genotypic determination of resistance profiles in *Klebsiella* spp. uropathogens

#### Antibiogram

Antibiotic susceptibility was assessed using the ATB Ur systems and the automated VITEK^®^ 2 Compact system. Inoculi of 0.5 McFarland opacity were prepared. These were then inoculated into the cups of the ATB Ur systems and incubated aerobically at 37 °C for 18–24 h. AST-N antibiogram cards for the VITEK^®^ automated system were also used to assess the susceptibility profiles.

### Molecular research into carbapenem and fosfomycin resistance genes

Genotypic characterization of the resistance of *Klebsiella* uropathogen to last-line therapeutic molecules was carried out on 22 strains using conventional PCR. The plasmid gene sequences for resistance to carbapenems (*bla*_IMP_, *bla*_KPC_ and *bla*_VIM_) and fosfomycin (*fos*A, *fos*B and *fos*C) and the chromosomal genes for fosfomycin only (*glpT* and *uhpT*) were amplified using specific primers described in previous studies [19–22]. Reaction mixtures of 20 µl in total consisted of 18 µl of PCR mix (1× master mix, 1 pmol primer pair, 1.5 µM MgCl2 and ultrapure water) and 2 µl of DNA from *Klebsiella* spp*.* strains. Amplification was carried out in a thermal cycler (Bio-Rad, T100^™^, USA) using the amplification conditions for *bla*_IMP_, *bla*_KPC_ and *bla*_VIM_: an initial denaturation (95 °C/15 min), followed by 34 cycles of denaturation (94 °C/5 s), annealing (57 °C/1 min) and extension (72 °C/1 min) with a single final extension (72 °C/5 min). For *fosA*, *fosB*, *fosC*, *glpT*, and *uhpT*, the following amplification conditions were used: an initial denaturation (95 °C/15 min), followed by 34 cycles of denaturation (94 °C/5 s), annealing (55 °C/20 s), and extension (72 °C/2 min) with a final extension (72 °C/10 min).

### Study of the polymorphism of isolated *Klebsiella* spp. strains

The genetic diversity of *Klebsiella* spp*.* uropathogens was screened using the ERIC-PCR method on 22 strains. The ERIC primer sequences used were 5′-ATGTAAGCTCCTGGGGATTCAC-3′ and 5′-AAGTAAGTGACTGGGGTGAGCG-3′ for the forward and reverse primers, respectively [20]. ERIC sequences were amplified in a final reaction volume of 20 µl containing 2 µl of total DNA diluted 1 : 10, 10 µl of 1× master mix AmpliTaq Gold 360 Master Mix (Thermo Fisher Scientific), 2 µl of forward and reverse primers (1 pmol), 1.5 mM MgCl_2_ and ultrapure water. Amplification was carried out in a thermal cycler (Bio-Rad, T100^™^, USA), using the following conditions and programme: an initial denaturation (95 °C/5 min), followed by 30 cycles of denaturation (94 °C/1 min), annealing (52 °C/1 min) and extension (72 °C/2 min) with a single final extension (72 °C/5 min). The ERIC-PCR profiles were analysed visually by comparing the amplified DNA bands. Each band corresponds to a specific genomic fragment, and the banding pattern reflects the ERIC-PCR profile of the isolate. The unweighted pair group method with arithmetic mean (UPGMA) was used to analyse the sequences obtained for the ERIC-PCR using the online data analysis service (http://insilico.ehu.eus/dice_upgma/), allowing the construction of the dendrogram by grouping strains according to their banding pattern similarities.

### Migration and visualization on agarose gel

PCR products were separated by electrophoresis on a 1.5% agarose gel immersed in an electrophoresis tank containing 1× Tris-borate-EDTA (TBE). Finally, a mixture containing 8 µl of amplicons and 5 µl of loading blue was inoculated into the wells. Migration was performed using a generator at 170 volts and 210 milliamperes for 1 h. Bands were visualized using a transilluminator, an optical system coupled to a computer running Quantum software.

### Statistical analyses

Statistical data were collected and processed using Microsoft Excel Professional 2016. They were then analysed using RStudio version 3.0. The chi-square test was used to compare numbers and prevalences in order to determine whether their distribution was homogeneous in the population, at a significance level of 5%. Exploratory correspondence factorial analyses were carried out using the R software package to describe potential spatial associations between the diversity of ERIC-PCR banding patterns and antibiotic resistance. Proximity between variables in the factorial plane was interpreted as a descriptive trend, where variables close to each other suggested a possible pattern of occurrence, while those far apart suggested an absence of such a trend.

## Results

### General presentation and socio-demographic parameters and prevalence of UTI

A total of 343 patients admitted to the HIAOBO as in-patients and/or out-patients for urine cytobacteriological examinations were recorded during the study period. Of these patients, 60.64% (208 out of 343) were women. Non-pregnant women were the most representative population with 88.46% (184 out of 208), while pregnant women represented 11.54% (24 out of 208) with a *P*-value=2.2.10^−16^. The prevalence of UTI in women was 43.75% (91 out of 208), with 58.33% (14 out of 24) in pregnant women and 41.85% (77 out of 184) in non-pregnant women, with a non-significant difference (*P*-value=0.1894). The various socio-demographic parameters characteristic of the female population are shown in Table 1.

**Table 1.** Socio-demographic parameters of the study

Parameter	Non-pregnant women *N*=**184**	Pregnant women *N*=**24**
	N (%)	N (%)
**Marital status**		
Single	90 (48.91)	6 (25)
Cohabitation	49 (26.63)	13 (54.17)
Married	35 (19.02)	5 (20.83)
Widow	10 (5.44)	0 (0.00)
**Ages**		
[1–18]	15 (8.15)	0 (0.00)
[18–45]	123 (66.85)	23 (95.83)
[45–81]	46 (25)	1 (4.17)
**Level of study**		
Primary	17 (9.24)	0 (0.00)
Without	17 (9.24)	0 (0.00)
Secondary	95 (51.63)	14 (58.33)
University	55 (29.90)	10 (41.67)
**Professional situation**		
Employed	72 (39.13)	4 (16.67)
Unemployed	112 (60.87)	20 (83.33)
**UTI**	**77 (41.85)**	**14 (58.33)**

The analysis of Table 1 shows that single non-pregnant women (48.91%) and pregnant cohabiting women (54.17%) are the most represented in this study. The average age was 36±16 years, with the most represented age group being 18 to 45 years (60.58%), representing 66.85% of non-pregnant women and 95.83% of pregnant women. Secondary education was also the most prevalent, with prevalence rates of 51.63% (95 out of 184) and 58.33% (14 out of 24) for non-pregnant and pregnant women, respectively.

### Distribution of germs

The involvement of *Klebsiella* uropathogens in women’s UTIs was 29.03% (27 out of 93) compared with 53.76% (50 out of 93) for other enterobacteria, followed by non-fermentative bacilli (9.68%), Gram-positive cocci (4.3%) and yeasts (3.2%) (Table 2). The specific richness of *Klebsiella* was three species. *Klebsiella pneumoniae* was the most abundant species with a rate of 70.4% (19 out of 27), followed by *Klebsiella aerogenes* with 22.2% (6 out of 27) and *Klebsiella oxytoca* which was the least represented species with 7.41% (2 out of 27). Depending on the target population, two and one species of *K. pneumoniae* and *K. aerogenes*, respectively, were isolated from pregnant women, compared with non-pregnant women, where all three species were found, with *K. pneumoniae* dominating (70.83%) (Table 2).

**Table 2.** Distribution of isolated uropathogens

	**N=93**	Non-pregnant women N=**77**	Pregnant women N=**16**
	n (%)	n (%)	n (%)
***Klebsiella*** **spp.**	**27 (29.03)**	**24 (31.17)**	**3 (18.75)**
*K. aerogenes*	6 (22.22)	5 (20.83)	1 (33.33)
*K. pneumoniae*	19 (70.37)	17 (70.83)	2 (66.67)
*K. oxytoca*	2 (7.41)	2 (8.34)	0 (0.00)
**Other enterobacteria**	**50 (53.76)**	**41 (53.25)**	**9 (56.25)**
**Non-fermentative bacilli**	**9 (9.68)**	**6 (7.79)**	**3 (18.75)**
**Gram-positive bacteria**	**4 (4.30)**	**3 (3.89)**	**1 (6.25)**
**Yeast**	**3 (3.22)**	**3 (3.89)**	**0 (0.0)**

### Phenotypic prevalence of *Klebsiella* spp. uropathogenic resistance to last-line antibiotics

The resistance profiles of the 27 strains of *Klebsiella* spp*.* to the 4 classes of antibiotics are presented in Table 3. Analysis of Table 3 revealed that the rate of uropathogen resistance to last-line therapeutic molecules varied according to the antibiotic. It appeared that the resistance rate to third-generation cephalosporins (C3G) was high (55.56%), with 59.26% of strains resistant to cefoxitin, 55.56% to cefotaxime and 51.85% to ceftazidime. Similarly, these bacteria also had high levels of resistance to fourth-generation cephalosporins (C4G), including cefepime, with a rate of 40.74% and fosfomycin (40.74%). Furthermore, the prevalence of resistance to third-generation fluoroquinolones (F3G) averaged 25.92% and was found to be low for carbapenems (7.41%).

**Table 3.** Rates of resistance to last-line antibiotics

Bacteria	Number (N)	C3G			C4G	F3G	Carbapenem	Fosfomycin
		Cefotaxime n (%)	Cefoxitin (%)	Ceftazidime n (%)	Cefepime (%)	Levofloxacin (%)	Imipenem (%)	Fosfomycin (%)
*K. aerogenes*	**6**	4 (66.67)	4 (66.67)	3 (50.00)	3 (50.00)	2 (33.33)	0 (0.00)	2 (33.33)
*K. oxytoca*	**2**	1 (50.00)	2 (100.00)	1 (50.00)	1 (50.00)	0 (0.00)	0 (0.00)	1 (50.00)
*K. pneumoniae*	**19**	10 (52.63)	10 (52.63)	10 (52.63)	7 (36.84)	5 (26.31)	2 (10.53)	8 (42.10)
**Global prevalence of resistance**	**27**	**15 (55.56)**	**16 (59.26)**	**14 (51.85)**	**11 (40.74)**	**7 (25.92)**	**2 (7.41)**	**11 (40.74)**
**Average resistance rate**		**15 (55.56)**			**11 (40.74)**	**7 (25.92)**	**2 (7.41)**	**11 (40.74)**

### Molecular characterization of the resistance genes screened

Molecular screening for resistance genes was performed on 22 strains of *Klebsiella* spp*.* and recorded a detection prevalence of 81.82% (18 out of 22). The results reveal a strong presence of the *bla*_KPC_ gene (40%), followed by the *fos*A gene (36%) and *glpT* (20%). A low presence of the *uhpT* gene (4%) was recorded, with no detection of the *fos*B, *fos*C, *bla*_IMP_ and *bla*_VIM_ genes. In addition, the results showed that mono-detection of resistance genes was most common (61.1%; *n*=11/18) compared with co-detection (38.9%; *n*=7/18). The prevalence of mono-detection of plasmid genes was 81.8%, with the KPC gene dominating (66.7%). The most representative co-detections were the *fos*A-KPC association (42.9%), followed by the *fos*A-*glp*T association (28.6%).

### Molecular diversity of *Klebsiella* spp. isolates based on ERIC-PCR

**Fig. 1.**
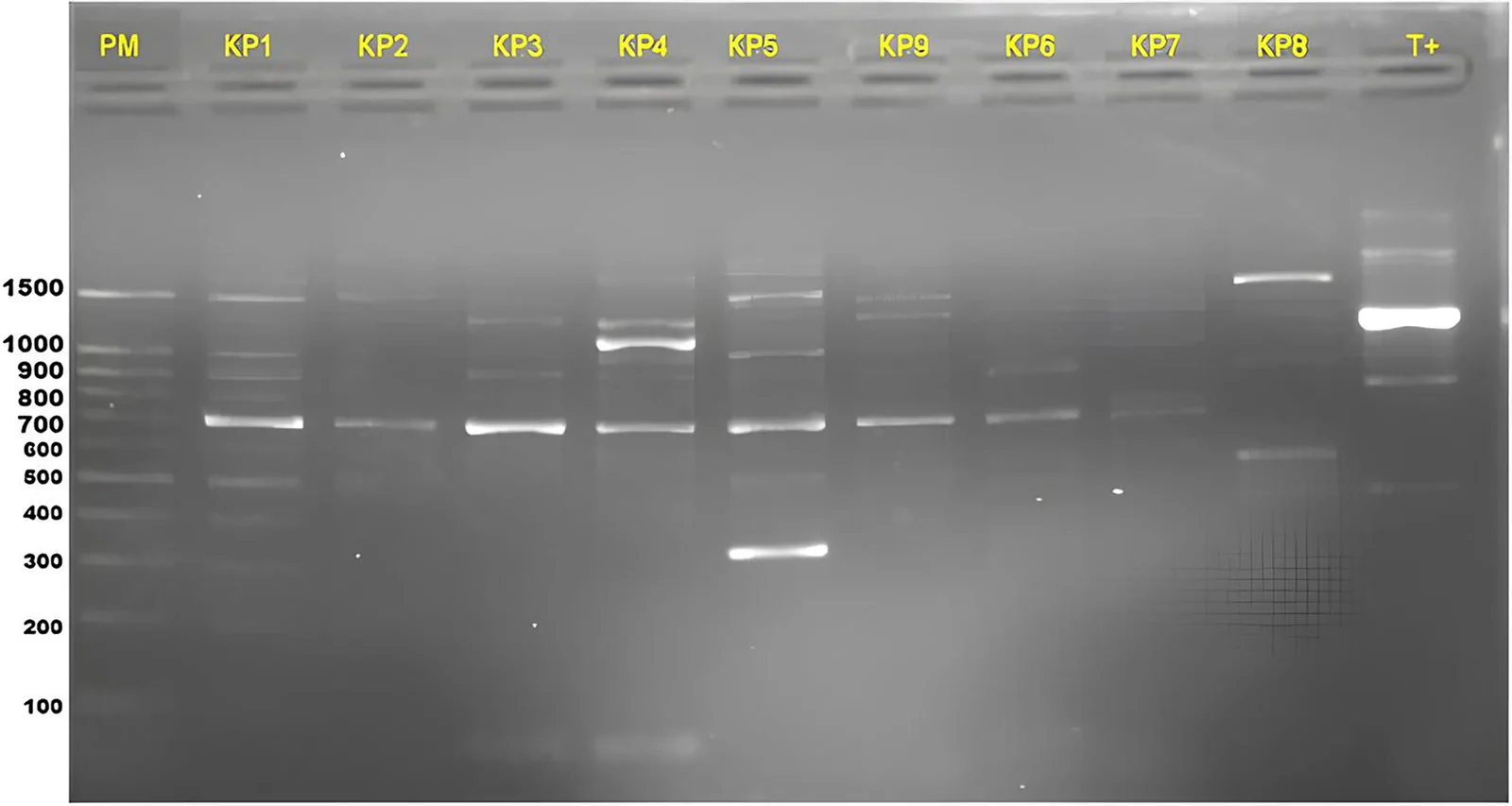
ERIC-PCR profiles of *Klebsiella* spp*.* on 1.5% electrophoretic gel. PM, molecular weight marker (1,500 bp); T+, positive control; KP, *K. pneumoniae*.

Molecular typing of the 22 *Klebsiella* spp. isolates by ERIC-PCR was successful for all strains (100%, 22 out of 22), yielding various banding patterns as shown in Fig. 1.

The results showed DNA banding patterns ranging from 1 to 10 bands with sizes between 50 and 1,800 bp (Fig. 1). The most frequent bands were 900 and 700 bp, and the least frequent were 500, 400, 300, 200, 50 and 300 bp. Profile identification shows 19 distinct ERIC-PCR profiles with 17 non-repeated and 2 repeated profiles (Table 4).

**Table 4.** Profile of genetic fingerprints obtained from strains of *Klebsiella* spp.

Strain	Band diversity	Minimum band	Maximum band	Dominant band (%)	No. of profile	Non-repeated profile	Repeated profile	No. of cluster
22	27	50	1,800	700 (95.5)	19	17	2	9
				900 (31.80)				

Twenty-seven bands were found within the *Klebsiella* strains, with 95.5% of bands at 700 bp being representative compared with 31.80% of bands at 900 bp.

**Fig. 2.**
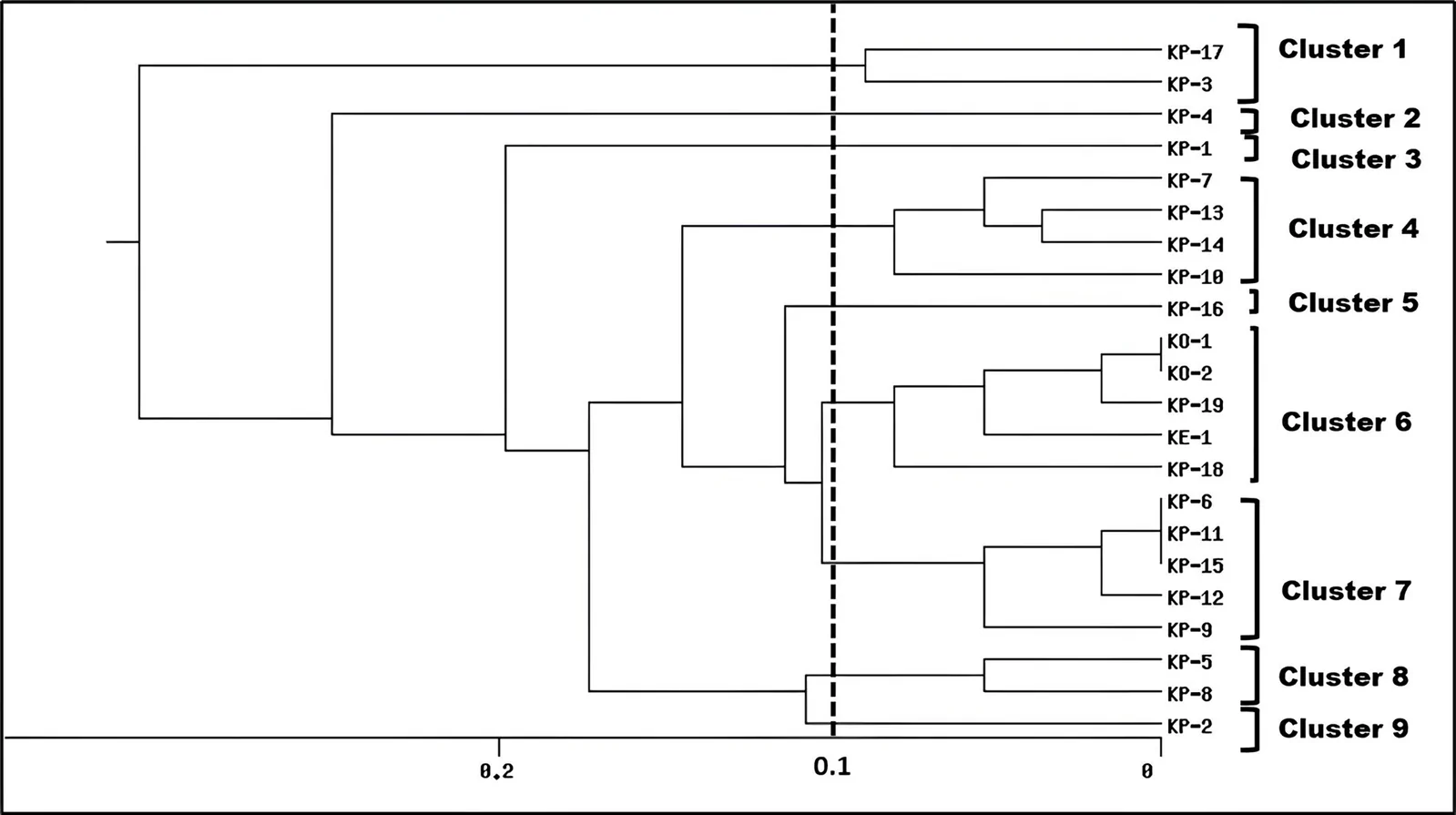
Dendrogram of *Klebsiella* spp*.* strains generated using UPGMA.

Analysis of the dendrogram shows that ERIC-PCR categorizes the 22 strains of *Klebsiella* spp. into 9 clusters based on banding profile similarities (Fig. 2).

Analysis of Fig. 2 highlighted that several strains exhibit indistinguishable or highly similar ERIC profiles. Specifically, two strains from cluster 6 (KO-1 and KO-2) and three strains from cluster 7 (KP-6, KP-11 and KP-15) showed high similarity patterns, suggesting a potential similar ERIC-PCR profiles.

### Relationship between ERIC-PCR molecular profiles and resistance patterns in *Klebsiella* spp.

Comparison between the different phenotypic and genotypic profiles of *Klebsiella* strains revealed a concordance rate of 36.36% (8 out of 22) against 63.64% (14 out of 22) of discordance between these 2 methods. The characteristics of each strain are shown in Table 5.

**Table 5.** Antibiotic-resistant characteristics of *Klebsiella* strains

Strain	Phenotypic resistance					Molecular resistance							Cluster
	C3G	C4G	F3G	Carba	FOS	Carbapenemase	Fosfomycin gene				Carbapenemase/fosfomycin		
						KPC	Fos A	*glpT*	*fosA*-*glpT*	*fosA*-*uhpT*	KPC-Fos A	KPC-GlpT	
*K. pneumoniae 17*	R	R	S	R	R						P		**1**
*K. pneumoniae 3*	R	R	S	S	S	P							
*K. pneumoniae 4*	S	S	S	S	S	P							**2**
*K. pneumoniae 1*	R	R	S	S	S							P	**3**
*K. pneumoniae 7*	R	R	R	S	R								**4**
*K. pneumoniae 13*	S	S	S	S	S			P					
*K. pneumoniae 14*	R	R	S	S	R								
*K. pneumoniae 10*	S	R	S	S	S						P		
*K. pneumoniae 16*	S	S	S	S	S	P							**5**
*K. oxytoca 1*	R	R	S	S	S								**6**
*K. oxytoca 2*	S	S	S	S	R	P							
*K. pneumoniae 19*	S	S	S	S	R	P							
*K. aerogenes*	R	R	S	S	S	P							
*K. pneumoniae 18*	S	S	R	S	R				P				
*K. pneumoniae 6*	R	S	R	S	R								**7**
*K. pneumoniae 11*	R	S	S	S	R								
*K. pneumoniae 15*	S	S	S	S	S								
*K. pneumoniae 12*	R	R	R	S	R		P						
*K. pneumoniae 9*	R	S	S	S	S						P		
*K. pneumoniae 5*	S	R	S	S	S		P		P				**8**
*K. pneumoniae 8*	R	S	S	S	S			P					
*K. pneumoniae 2*	S	S	S	S	S		P			P			**9**

Carba, carbapenem; FOS, fosfomycin; P, positive; R, resistant; S, sensitive.

Compared to the concordance of the profiles obtained, 37.5% (3 out of 8) showed an absence of both phenotypic and molecular resistance (*K. pneumoniae* 15, 16 and *K.* oxytoca 1), while 62.50% of *Klebsiella* strains showed phenotypic and genotypic resistance. *K. pneumoniae* strains 6, 11 and 12 showed phenotypic resistance to fosfomycin, which was confirmed genotypically by the presence of the *fos*A gene. *K. pneumoniae* 18, which had the same phenotypic profile, also possessed the *fos*A and *glp*T genes. *K. pneumoniae* 17 had phenotypic resistance to imipenem and fosfomycin, confirmed by the detection of the KPC and *fos*A genes in the latter (Table 5). With regard to discordant profiles, 71.42% (10 out of 14) of *Klebsiella* spp*.* strains were sensitive to antibiotics and possessed resistance genes, of which 30% (3 out of 10) had the KPC gene (*K. pneumoniae* 3, 4, *K. aerogenes*), 20% had the *glp*T gene (*K. pneumoniae* 8, 13) and the KPC and *fos*A genes (*K. pneumoniae* 9, 10) and 10% had both *fos*A and *glp*T (*K. pneumoniae* 1) and KPC and *glp*T (*K. pneumoniae* 5). Also, 14.29% (2 out of 14) of *Klebsiella* strains showed a phenotypic resistance profile to the majority of antibiotics, but no resistance gene was present in these strains (*K. pneumoniae* 7, 14). In addition, 14.29% (2 out of 14) showed other discordant profiles: *K. pneumoniae* 9 and *K. oxytoca* 2 were resistant to fosfomycin but carried the *KPC* gene without the screened fosfomycin genes (Table 5).

**Fig. 3.**
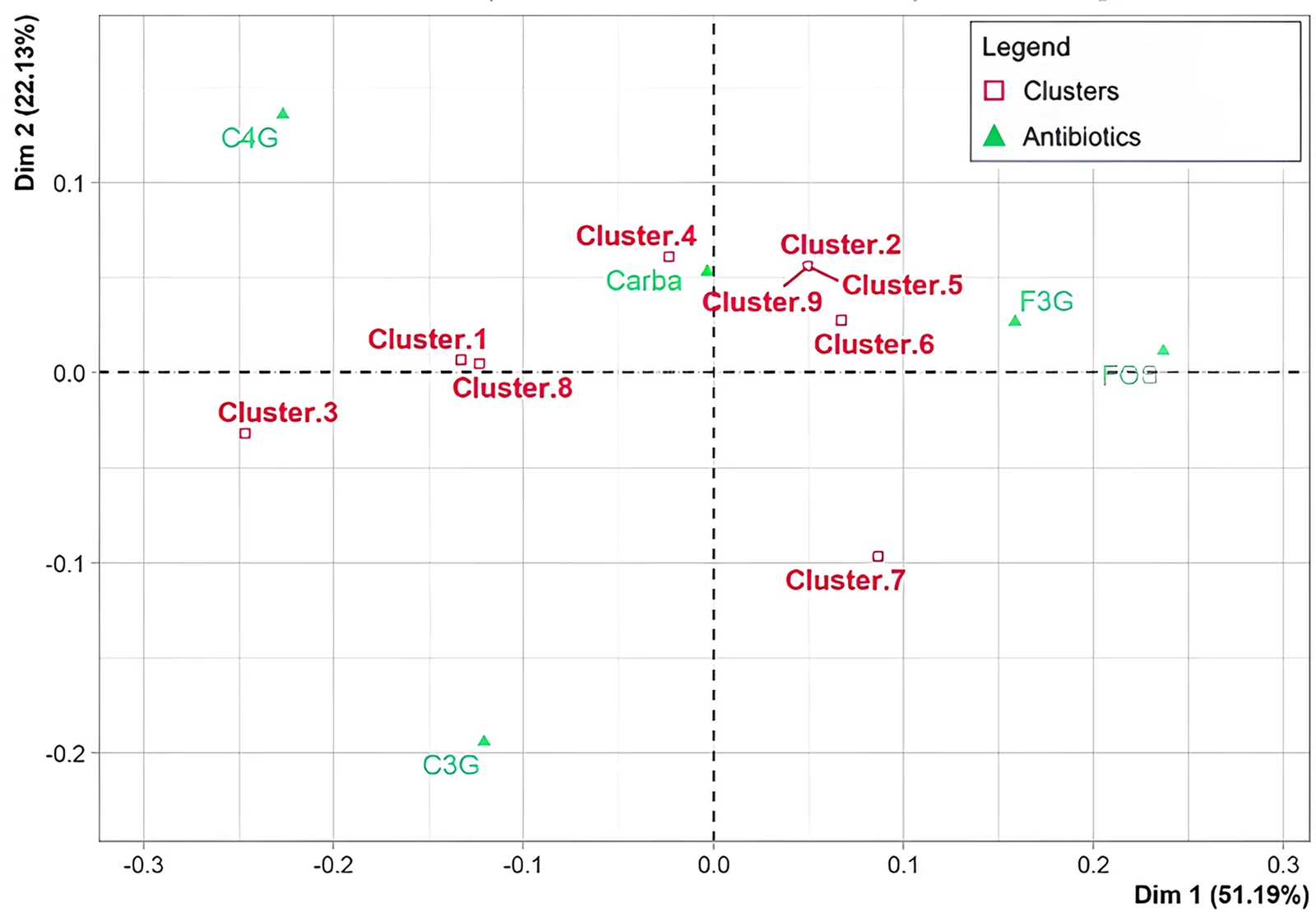
Projections on the Dim1/Dim2 plane calculated from factor analysis of the correspondences (AFC) of the nine bacterial clusters and antibiotic resistance profiles.

**Fig. 4.**
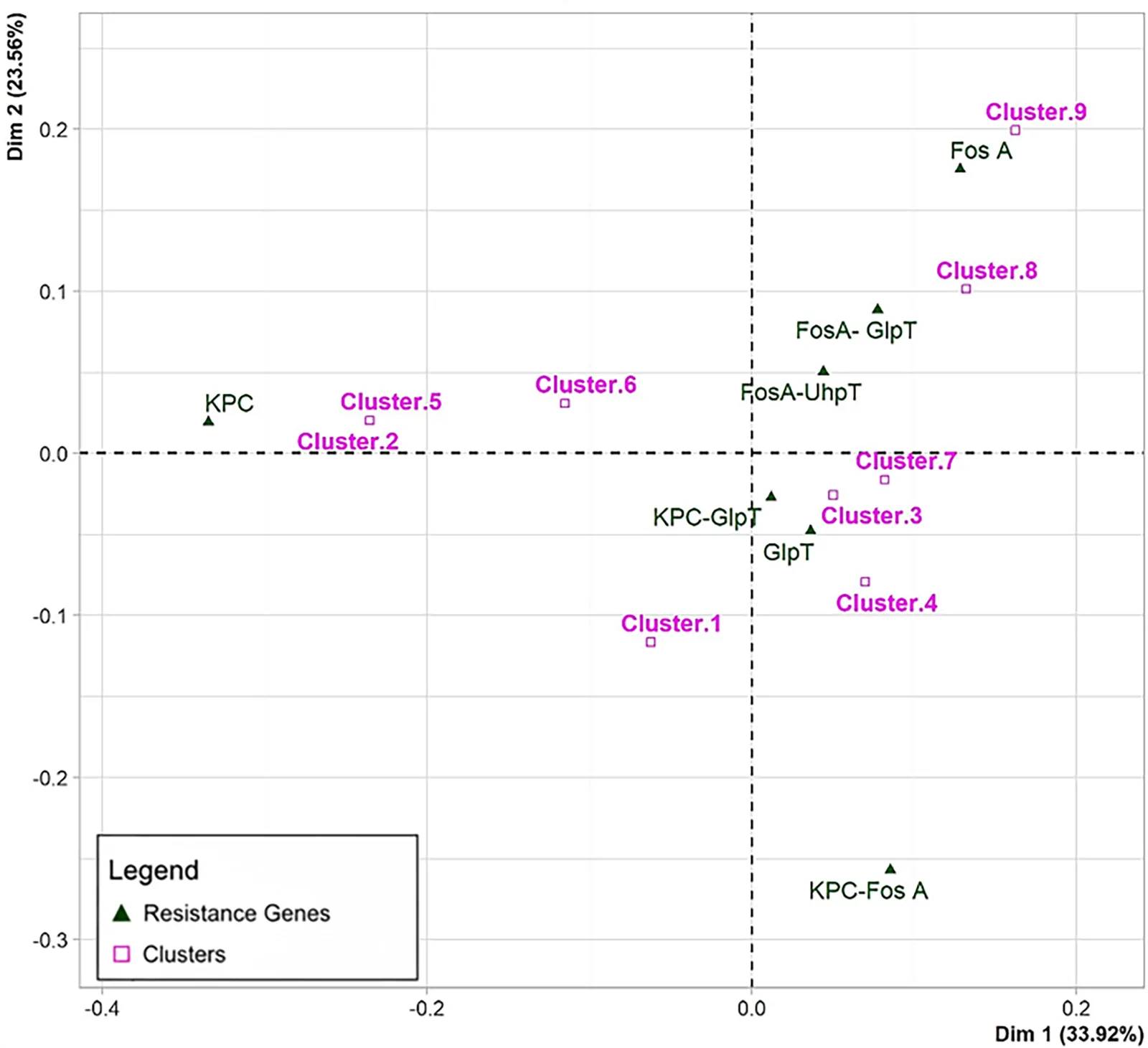
Projections on the Dim1/Dim2 plane calculated from factor analysis of the correspondences (AFC) of the nine bacterial clusters and antibiotic resistance profiles.

Furthermore, an exploratory Correspondence Factorial Analysis was conducted to describe potential trends between ERIC-PCR clusters and antibiotic resistance profiles, as well as with the screened resistance genes. The spatial distribution and proximity of these variables on the factorial plane are illustrated in Figs 3 and 4.

The positioning of variables on the factorial plane illustrates exploratory descriptive patterns between the studied categories. Fig. 3 captures 73.32% of the total inertia, indicating that the first two dimensions provide a representative summary of the variance within this specific dataset. Along the Dim 1 axis (51.19%), the analysis reveals a spatial separation between cluster 4 and carbapenem resistance, illustrating their distinct positioning in the factorial space. Conversely, a relative spatial proximity is observed between clusters 2, 5, 6 and 9 and resistance to both C3G and carbapenems, suggesting a potential descriptive trend towards their co-occurrence on the plane. Finally, cluster 7 is positioned near the origin, indicating that, in this exploratory context, it does not contribute significantly to the variations in resistance profiles captured by these dimensions.

Fig. 4 accounts for 57.48% of the total inertia. In this exploratory analysis, the first dimension plane (Dim 1, 33.92%) displays descriptive patterns between clusters and resistance genes. A spatial separation is observed between clusters 2, 5 and 6 and the projection of the *KPC* gene. In contrast, a relative graphical proximity is noted between clusters 3, 4 and 7 and the presence of the *glpT* gene, as well as the *KPC-glpT* co-detection. This proximity reflects a possible descriptive trend on the factorial plane for these determinants within this specific dataset. Finally, the *KPC-fosA* co-detection projects in an isolated manner, indicating that no specific pattern is perceptible with the studied clusters on this exploratory factorial plane.

## Discussion

The aim of this study was to show the involvement of *Klebsiella* strains in UTIs in women and to characterize their resistance to last-line therapeutic molecules, as well as their ERIC-PCR profiling diversity.

An overall prevalence of UTI of 35.86% was recorded at HIAOBO in Libreville, Gabon, in this study. This rate is higher than that recorded in Franceville (29.2%) [4]. However, it remains lower than that obtained by Mouanga-Ndzime *et al.* in the same locality (59%) [23]. These differences in prevalence can be explained, on the one hand, by the difference in the bacterial culture methods used and, on the other hand, by the study population. These two studies used the culture method of inoculating 10 µl of urine onto agar media, unlike our study, which used Urilines. Also, the work of Mouanga-Ndzime *et al.* focused on children aged 0–17 months, unlike our study, which was not selective in terms of patient type. Obviously, many studies, such as Hudson *et al.*’s, have shown that children are more prone to UTIs than adults [24].

Specifically, the prevalence of UTIs among women in this study was 43.75%. This is higher than that described in some studies, notably in Mali, where it was 28.35%, and 15.37% in Ethiopia. It is lower than the 64.4% prevalence reported in the work of Samje M *et al.* in Cameroon [25–27]. The high prevalence of UTIs in women may be explained by female anatomy and physiological changes. The shorter urethra in women than in men, and the proximity of the urinary meatus to the anus, favours contamination by faecal flora and encourages the development of UTIs [28, 29].

The isolation and identification of uropathogens in women in this study show the involvement of *Klebsiella* spp*.* strains in 27.45% of UTIs, and they represent the second most common uropathogen. The mean prevalence obtained is similar to that recorded in the work of Kaduma *et al.* (23%). However, it is higher than the rates of *Klebsiella* spp*.* isolation in studies by Ana Flores-Mireles (10.1%) and Tekkarışmaz *et al.*, conducted in Europe and Asia, where *Klebsiella* spp*.* was the second uropathogen (8%) [30, 31]. The position of *Klebsiella* spp*.* strains as the second uropathogen in female and human UTIs, in general, may be explained by the high expression of virulence genes encoding type 1 pilus, which are involved in the process of adhesion and colonization of the urinary tract by this uropathogen. In addition, *Klebsiella* spp*.* has an arsenal of pathogenicity and a great capacity to form biofilms, which are properties that enable it to strongly resist the body’s immune defences, to reinfect and finally to evolve in the urinary tract [32].

The *Klebsiella* spp*.* strains listed as members of the ESKAPE group are identified as bacteria likely to present high rates of resistance to antibiotics. Indeed, assessment of the prevalence of antibiotic resistance in this study shows that uropathogenic *Klebsiella* spp*.* strains have high rates of resistance to C3G (56%), C4G (40%), fosfomycin (40%) and average rates of resistance to F3G (24%). These recorded resistance profiles are very high compared with those obtained between 2016 and 2018 by Dikoumba *et al.*, during a multicentre study carried out in seven major towns in Gabon. This study revealed that *Klebsiella* spp*.* strains had resistance rates to C3G, C4G, F3G and carbapenems of 10.33, 9.25, 7.5 and 3.5%, respectively [33]. However, our results seem to be similar to those reported in recent studies [34, 35]. This sharp increase in resistance among these bacteria in the same area and in such a short space of time highlights the need to monitor the distribution of resistance genes in *Klebsiella* spp. and to implement appropriate policies to reduce this phenomenon, which is leading us inexorably towards a return to the post-antibiotic period.

Molecular screening of *Klebsiella* uropathogen resistance was performed in this study and revealed the predominance of the *bla*_KPC_ (40%) and *fos*A (36%) genes. Also, co-expression of these two genes was found in 42.9% of strains. Higher rates of detection for these genes have been reported in previous studies, with over 90% detection, respectively, for *fos*A in the USA and *bla*_KPC_ in China [36, 37]. However, the predominance of the *bla*_KPC_ gene can be explained by the fact that it is the most clinically important serine carbapenemase and is frequently observed in *Klebsiella* isolates associated with infections such as UTIs, septicaemia and pneumonia [38]. Furthermore, the absence of *bla*_IMP_, *bla*_VIM_ and fosfomycin resistance genes, namely *fos*B and *fos*C in this study, could be due to the presence of other resistance genes in these bacteria not included in our restricted PCR panel, in particular the *bla*_OXA_ gene for resistance to carbapenems and *Mur*A for resistance to fosfomycin. It can also be explained by the presence of other resistance mechanisms, such as the presence of efflux pumps. In addition, the presence of the *bla*_KPC_ gene in enterobacteria induces resistance not only to carbapenems but also to other classes of antibiotics through the phenomenon of cross-resistance [39]. The *fos*A3 gene is found on the same transposon as *bla*_KPC_ and is thought to modulate the *bla*_KPC_ gene, leading to resistance to fosfomycin, as described by Li *et al.* [40]. Many studies have highlighted the potential link between phenotypic and molecular resistance [41, 42].

However, the relationship between molecular resistance and the associated phenotypic changes is complex and not yet fully understood. Our study revealed a high discordance rate (71.42%) between phenotypic and genotypic profiles. Similar results were also observed in a study of carbapenem resistance in uropathogens, in which 66 carbapenem-resistant isolates had none of the carbapenem resistance genes tested [35]. This high level of discordance highlights the inherent difficulty of predicting resistance phenotypes from a restricted PCR gene panel.

Methodologically, the limited number of resistance genes screened in this study means that other untested genes or alleles could be responsible for the observed resistance. Biologically, phenotypic resistance in the absence of the targeted genes may be explained by mechanisms not assessed in our panel, such as the loss or downregulation of porins or the overexpression of multidrug efflux pumps, which are common in *K. pneumoniae* [43, 44]. For fosfomycin, resistance can also arise from mutations in chromosomal genes like *murA* or regulatory genes that were not sequenced here. Conversely, the detection of resistance genes in phenotypically susceptible isolates may result from low gene expression, regulatory effects or the genes being ‘silent’ under standard laboratory testing conditions. Therefore, our molecular results should be interpreted as a partial snapshot of the resistance landscape rather than a complete explanation of the observed phenotypes. Finally, the specific ecological and epidemiological context of Gabon should be taken into account. This low concordance suggests a local adaptation of strains to unique environmental selective pressures. Therefore, although molecular tools are rapid, the low concordance observed here indicates that genotype alone cannot yet reliably predict phenotype in this particular ecological niche. This highlights the importance of systematically combining phenotypic and molecular data to achieve a more robust assessment of resistance profiles.

The results of molecular screening of uropathogenic *Klebsiella* strains using ERIC-PCR revealed 19 different banding patterns, divided into 9 unique clusters, with sizes ranging from 50 to 1,800 bp, indicating molecular heterogeneity in these strains. This size variability is comparable to that obtained in other studies. Indeed, *Klebsiella* spp*.* strains isolated from hospital infections in some studies presented profiles with bands ranging from 100 bp to more than 1,500 bp. Also, the molecular diversity of *Klebsiella* spp*.* clinical isolates has already been reported in the literature. This is the case in Egypt, where out of 100 typed *Klebsiella*, 57 presented different profiles arranged in 3 clusters [45]. Similarly, in Iran, Mehr *et al.* showed that 35 clinical isolates of *Klebsiella* spp*.* presented 32 different ERIC profiles [46]. This diversity of ERIC sequences seems to be linked to the appearance of mutations over time, particularly deletions and insertions driven by genomic adaptation to environmental pressures. Consequently, this results in isolates with distinct molecular signatures compared to ancestral strains [47].

In this study, several *Klebsiella* spp*.* isolates exhibited closely related ERIC-PCR banding patterns. Similar observations have been reported globally; for instance, in China, identical profiles were identified in strains from frozen poultry [48]. And in Iran, 10.5% of typed isolates shared matching patterns [49]. While such molecular similarity may suggest potential similarities in the distribution of profiles, these results must be interpreted with caution. The clustering of isolates (KO1-KO2 and KP6-KP11-KP15) among women in the 20–35 age group raises questions regarding possible descriptive trends. While transmission of uropathogens through shared environments or sexual contact has been suggested by the World Health Organization [50], the current data from ERIC-PCR do not allow for a definitive epidemiological link. We acknowledge that ERIC-PCR has inherent limitations, including moderate discriminatory power and variability in reproducibility, which may lead to overestimating similarity between isolates. Unlike 'gold-standard' methods such as PFGE, multilocus sequence Typing (MLST), or whole-genome sequencing (WGS), ERIC-PCR provides a preliminary screening rather than definitive confirmation of clonal relationships or transmission. Therefore, the clusters identified here should be viewed as suggestive of potential trends that require further validation through high-resolution genomic tools.

Numerous studies have explored the spatial proximity between the molecular profiles of bacterial isolates and the variability of resistance profiles. In theory, sharing similar ERIC-PCR banding patterns belonging to the same cluster should exhibit similar resistance phenotypes. This study is one of the first to explore, through exploratory Correspondence Analysis, the spatial patterns between ERIC-PCR banding patterns and resistance profiles in *Klebsiella* spp*.* isolates from Gabon.

Although exploratory correspondence factorial analysis reveals spatial groupings suggesting possible co-occurrence trends between certain ERIC clusters and specific resistance profiles, our overall results show that genetic diversity, as defined by ERIC typing, does not constitute a rigid predictor of resistance. The correspondence factorial analysis used here is a descriptive method aimed at visualizing spatial structures; therefore, it does not allow for the establishment of strict statistical causal links. This lack of strict correlation is also explained by the very nature of ERIC sequences, which are intergenic imperfect palindromes whose functions do not appear to be linked to fundamental mechanisms of survival or resistance [18]. Indeed, although ERIC sequences are the subject of numerous studies, their origins and potential functions remain poorly understood. Most short repetitive bacterial sequences are imperfect palindromes capable of forming secondary structures, thereby improving mRNA stability. Conversely, the majority of these elements may be merely non-functional waste [17]. This suggests that the functions of these elements do not involve fundamental aspects of bacterial growth or survival, and even less so bacterial replication.

This study, however, has several limitations. First, the relatively short sampling period combined with a modest sample size limits the scope of conclusions that can be drawn. Furthermore, the absence of environmental isolates hinders understanding of the ecological context and does not allow for assessing potential pathways for dissemination of resistance determinants. In addition, although molecular typing methods are commonly used to screen for similarities among isolates and sources of infection, it would be advisable that, before being used for those purposes, PCR methods for molecular typing require careful in-house validation of reproducibility and discriminatory power. This study highlights that while ERIC-PCR is useful for local screening, it lacks the resolution for definitive clonal analysis. Also, it would have been interesting to use MLST, PFGE or WGS, which seem to be more discriminatory than ERIC-PCR and are the gold-standard techniques.

## Conclusions

This study is one of the first to describe the diversity of ERIC-PCR profiles among *Klebsiella* spp*.* strains involved in UTIs in women in Gabon. The worldwide dissemination of antibiotic-resistant bacterial strains poses significant difficulties in clinical management in this context, and our work confirms that the female population is highly affected by UTIs. One of the main causal agents is *Klebsiella* spp., which is the second most common uropathogen, whose majority species is *K. pneumoniae*. This study also highlights the circulation of *Klebsiella* spp*.* multi-resistant to molecules of the latest therapeutic options. Molecular screening identified chromosomal and/or plasmid resistance genes that enable them to resist a very wide range of antibiotics, such as the *bla*_KPC_, *fos*A, *glp*T and *uhp*T genes. The analysis of the ERIC banding patterns reveals substantial genetic diversity among *Klebsiella* spp*.* isolates, while identifying with closely related profiles. The descriptive patterns observed between these ERIC-PCR profiles and antibiotic resistance phenotypes suggest that similar resistant strains may be circulating within the local population. Nevertheless, given the limited discriminatory power of ERIC-PCR, the restricted gene panel and the modest sample size, these findings should be interpreted with caution. They are more indicative of similarities in ERIC-PCR banding patterns than definitive evidence of clonality or epidemiological transmission. In this context, these preliminary findings underscore the need to strengthen local surveillance of *Klebsiella* spp*.*, not only in human infections but also in animals and the environment, in line with a ‘One Health’ approach that recognizes the interconnectedness of these reservoirs.

## Abbreviations

API: analytical profile index
C3G: third-generation cephalosporins
C4G: fourth-generation cephalosporins
CIRMF: Interdisciplinary Center for Medical Research of Franceville
ERIC-PCR: enterobacterial repetitive intergenic consensus PCR
F3G: third-generation fluoroquinolones
HIAOBO: Hôpital d’Instruction des Armées Omar Bongo Ondimba
MLST: multilocus sequence typing
TBE: tris-borate-EDTA
UPGMA: unweighted pair group method with arithmetic mean
UTI: urinary tract infection
WGS: whole-genome sequencing
